# Effects of the NiFe_2_O_4_ nanoadditive on the performance and emission characteristics of diesel engines: ultrasonic green synthesis by T3 hormone

**DOI:** 10.1039/d1ra04581d

**Published:** 2021-08-16

**Authors:** Samira Mandizadeh, Omid Amiri, Masoud Salavati-Niasari

**Affiliations:** Institute of Nano Science and Nano Technology, University of Kashan P. O. Box. 87317-51167 Kashan I. R. Iran salavati@kashanu.ac.ir +98 31 55913201 +98 31 55912383; Faculty of Chemistry, Razi University Kermanshah 6714414971 Iran; Department of Chemistry, College of Science, University of Raparin Rania Kurdistan Region Iraq

## Abstract

NiFe_2_O_4_ nanosheets were successfully synthesized *via* combined ultrasonic and combustion methods using triiodothyronine (T3) hormone as a biotemplate. Isodiesel and heavy diesel were selected as feedstocks to evaluate the ultrasound-assisted catalytic oxidation process. In this study, we focused on high performance of diesel engine with NiFe_2_O_4_ nanosheets. Various conditions such as catalyst dosage, hydrogen peroxide dosage, frequency range and catalyst morphologies of NiFe_2_O_4_ were investigated to achieve optimized conditions. High levels of sulfur compounds (98%) were removed using NiFe_2_O_4_ catalysts under determined conditions (1.0 g L^−1^ catalyst, O/S mole ratio = 2, frequency = 40 kHz and morphology of the nanocatalyst = nanosheets). The nickel ferrite nano additive was mixed with isodiesel and heavy diesel using an ultrasonicator device to achieve better stability. The results indicated that under the optimum amount (1% w/v), the NiFe_2_O_4_ nanostructure is the best additive to reduce NO_*x*_, CO, HC and smoke emission in diesel engines. Moreover, a change in the flash point and viscosity of diesel fuels was observed with the addition of nanosheets. NiFe_2_O_4_ could be recycled 3 times without a significant decrease in catalyst activity.

## Abstract 2

In the present study, nanocrystals are synthesized by the ultrasonic method in different morphologies with T3 hormone as a template. Different parameters are investigated for the synthesis of nanostructures.

## Graphical abstract

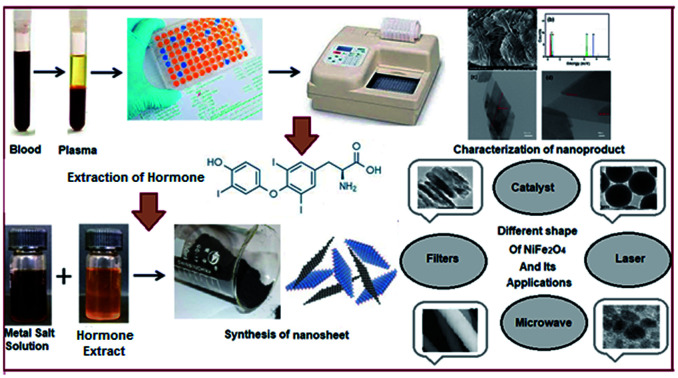

## 1. Introduction

Ferrite based nanomaterials with a spinel structure (AFe_2_O_4_ – A:Ni, Mg, *etc.*) are attractive candidates for a variety of applications such as micro-electronic/magnetic devices and microwave absorbers.^1,2^ Nickel ferrite (NiFe_2_O_4_) is one of the best spinel ferromagnetic oxides, where Ni^2+^ and Fe^3+^ ions occupy octahedral and tetrahedral sites, respectively.^3^ In general, the purity, physical properties and morphology of NiFe_2_O_4_ govern the performance of the system. For instance, nanocrystalline mesoporous structures with a pure phase are required for catalytic thermo-chemical hydrogen production, and gas sensing applications. To date, different shapes of NiFe_2_O_4_ nanostructures, such as sheets, fibers and rods, have been prepared using co-precipitation, sol–gel, reactive milling, solid state, micro-emulsion, sonochemical, and solvothermal methods. Recently, the ultrasonic method has attracted great attention due to its simplicity, high crystallinity of the product and size control. Among the different shapes of nanoferrite, NiFe_2_O_4_ with two-dimensional (2D) nanosheets provides a large number of accessible active sites, which may lead to good catalytic performance.^4–12^

Recently, utilization of non-toxic, biologically safe, and eco-friendly substances for the synthesis has attracted a lot of attention. The term “biomorphic mineralization” has been recently developed in order to attain nanomaterials with a desired morphology. In recent years, natural materials, like hormones, enzymes, and plant extracts, were applied to prepare nano-sized structures.^13^ However, among the natural materials, only few have shown technological interest briefly discussed. In nanomaterials production using biomorphic mineralization, the biotemplates can be used as reducing/capping agents during the reaction. These biotemplates are useful for synthesizing highly ordered morphologies and provide better properties of nanoferrites. Regeneration ability of the ferrites and performing the reactions in short times are important for industrial applications.^14^

Generally, diesel engines are called dirty engines. The control of pollutants in diesel fuel is important due to environmental concerns.^15–18^ The incorporation of new technologies in the field of chemistry has improved the efficiency of some chemical reactions. Hydrodesulfurization (HDS) is a new method for sulfur removal; unfortunately, this process requires high pressure and temperature.^19^ In recent years, catalytic oxidative desulfurization (CODS) has been developed as a new technology for deep desulfurization of diesel. Different oxidizing agents such as H_2_O_2_,^20^*t*-butyl-hydroperoxide^21^ and ozone^22^ have been utilized in this process. H_2_O_2_ is the mostly selected oxidant due to its environmental compatibility.^23^

Some techniques such as EGR (exhaust gas recirculation),^24^ water emulsion,^25^ and modifying the engine design^26^ have been considered to reduce NO_*x*_, HC and smoke emission from diesel engines. Several recent studies have shown that transition metals can be utilized as additives because these crystals help the combustion on the molecular level to achieve better emission reduction.^27,28^ Hence, NiFe_2_O_4_ nanosheets were selected as additives in this work. In this study, ultrasound waves were used to assist the ODS process. High local pressure and temperature can be produced by ultrasonic cavitation. They are very reactive and can oxidize sulfur compound to sulfide in fuel. Among the methods for desulfurization, ultrasound-assisted oxidative desulfurization (UAOD) is very important because it does not require expensive equipment and removes sulfur compound under mild conditions. Application of this catalyst in improving diesel quality can pass stringent laws may apply in future.^29–31^ Nanomaterials with desired morphologies are the best candidate for improving the catalytic activity. As mentioned before, the production routes/methods generally govern the morphology of the nanomaterials.^32^ Moreover, the catalytic performance of the materials also depends on their reaction conditions and the nature in the feed stream.^33^

Apart from various artificial organic templates, we are going to describe natural templates to prepare nanoferrite with improved chemical properties. In the present work, nanosheets of NiFe_2_O_4_ were synthesized *via* an ultrasonic assisted combustion method. To control the morphology of NiFe_2_O_4_, triiodothyronine (T3) hormone was used as a biotemplate. The nanosheet morphology of NiFe_2_O_4_ was used for catalytic oxidative desulfurization. Isodiesel and heavy diesel were selected as the feed to evaluate the catalytic activity. Other parameters such as NO_*x*_, CO, HC and smoke emission were evaluated by adding NiFe_2_O_4_ in the feedstock with an ultrasonicator device.

## 2. Experimental

### 2.1 Materials and method

The starting cationic sources Ni(NO_3_)_2_·*n*H_2_O (*M*_w_ = 290.79 g mol^−1^, mp = 56 °C, CAS no. 13478-00-7), Fe(NO_3_)_3_·9H_2_O (*M*_w_ = 404.00 g mol^−1^, mp = 47 °C, CAS no. 7782-61-8), lactose (C_12_H_22_O_11_, *M*_w_ = 342.30 g mol^−1^, CAS no. 63-42-3), and H_2_O_2_ (30% w/w, CAS no. 7722-84-1) were purchased from Sigma-Aldrich. Triiodothryonine horseradish peroxidase (T3-enzyme conjugate), tetramethylbenzidine (CAS no. 54827-17-7), H_2_SO_4_ (CAS no. 7664-93-9). All chemicals were supplied in analytical grade and utilized without further purification.

### 2.2 Synthesis of NiFe_2_O_4_ nanoparticles/nanosheets

T3 hormone was extracted on the basis product sheet provided by Leinco Technologies, Inc.^34^ Triiodothyronine (3,5,3′-triiodo-l-thyronine, T3) is produced by the thyroid gland. This hormone controls the body's diverse biochemical processes. T3 was extracted from the thyroid gland and secreted directly into the bloodstream based on the MICRO-EIA T3 test in a solid phase competitive enzyme immunoassay (EIA). In the T3 EIA, a second antibody (goat anti-mouse IgG) was coated on microtiter wells. The test sample was mixed with T3–enzyme conjugate in each well. After 15 min of incubation, the wells were washed to remove any unbounded T3–enzyme. An enzyme chromogen (hydrogen peroxide, H_2_O_2_, and tetramethylbenzidine, TMB) was added to the well to reflect blue color. To terminate the reaction, 1.0 M H_2_SO_4_ was added. The sign of reaction quenching can be clearly identified from color tone conversion (blue → yellow). The intensity of the yellow color is important to evaluate the concentration of free T3 in the sample.

Spinel NiFe_2_O_4_ was formulated and synthesized *via* a combustion method. Primarily, the metal nitrates were dissolved in distilled water in stoichiometric ratios. The aqueous solutions were added into the solution containing lactose and stirred for 30 min at 50 °C. Various ratios of lactose : nitrate were selected (0 : 1, 1.5 : 1, 3 : 1 and 6 : 1, samples 1–4) in order to obtain a pure and crystalline product with homogeneous morphologies.

**Fig. 1:**
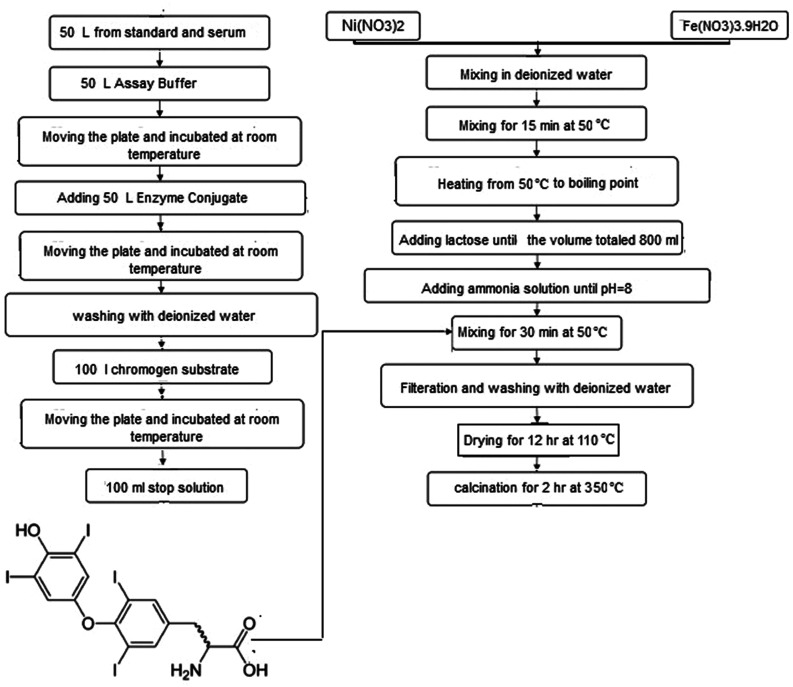
Preparation of the NiFe_2_O_4_ nanocatalyst.

Nanosheets particles were synthesized in the presence of 2 mmol T3 hormone. In a sample containing 6 molar ratio of lactose (sample 4 in this paper), an appropriate amount of T3 hormone (2 mmol) was added, stirred at 50 °C for 30 min and sonicated for 15 min. The obtained product was centrifuged for 10 min at 7000 rpm, decanted, and washed with distilled water and methanol. Finally, all the samples were calcined at 350 °C for 2 hours. A sample containing nitrates, and 6 molar ratio of lactose (sample 5) was synthesized without adding T3 hormone under the same experimental conditions. The preparation conditions are summarized in Table 1. Fig. 1 illustrates a flowchart of an implementation of a general representation of the nanocrystal production method.

**Table 1:** Preparation conditions, morphology, particle sizes and crystallite sizes of the as-prepared NiFe_2_O_4_ samples

Sample	Lactose : nitrate ratio	Morphology	Average particle size^a^ (nm)	Maximum average particle size (nm)	Crystallite size^b^ (nm)
Sample 1	0 : 1	Agglomerate	74–175	151–163	3.8
Sample 2	1.5 : 1	Semi-spherical	19–59	20–32	5.0
Sample 3	3 : 1	Agglomerate	71–153	68–75	16.7
Sample 4	6 : 1 + T3	Cuboid/plate	45–80 (height)	76–82	27.6
			234–568 (length)	284–291	
			142–377 (depth)	342–350	
Sample 5	6 : 1	Agglomerate	63–159	53–92	15.2

a Obtained from Fig. 2.

b Calculated from the Scherrer equation (Fig. 4).

### 2.3 Characterization

X-Ray diffraction (XRD) patterns were obtained from a diffractometer (Philips) with X'PertPro monochromatized Cu-Kα radiation. FESEM (Mira3 Tescan) was utilized to study the morphology of the products. The TEM images and HRTEM images were all obtained using a JEM2100 (JEOL Ltd, Japan). Energy dispersive spectrometry (EDS) analysis was performed using a Philips-XL30 microscope. FT-IR analysis was performed using a Nicolet Magna-IR, 550 spectrometer in KBr pellets. The magnetic properties of the prepared nanocatalysts were studied using a vibrating sample magnetometer (VSM, Meghnatis Kavir Kashan Co., Kashan, Iran). Thermal behavior of the synthesized nanocrystals was examined using a Thermogravimetric Analyzer model Schimadzu TGA-50H (Kyoto, Japan) from 20 °C to 400 °C. The Brunauer–Emmett–Teller (BET) method was used to determine the specific surface area.

### 2.4 Combustion tests

All combustion tests were performed at a constant engine speed (1500 rpm) and varying engine load ranging from 0 to 12 N m, at equal intervals of 3 N m. Air and fuel consumption rates were measured using an orifice meter (diameter of 20.6 mm) and flow meter sensor (Huba control type 680-out signal 0–10 volts direct current (VDC), accuracy ± 0.25% full scale (FS)), respectively. Cylinder pressure was measured using a Kistler piezoelectric pressure sensor of Model 6052C connected to a charge amplifier (Model GUNT CT100.13), while the engine speed was measured through a proximity sensor (WACHENDORFF of type PNP-N. O, Sn 4 mm, 10-30VDC, and 200 mA). A k-type thermocouple was applied to determine the exhaust gas temperature from the engine diesel after adding the catalyst in an amount of 1%. NO_*x*_, CO, HC and smoke emission were measured using an AVL Di-gas and AVL 437C, respectively. The feedstock was pumped to the engine diesel with a standard injection pressure of 201 bar and injection timing of 24° bTDC. Engine emissions were measured with an ECA 450 exhaust gas analyzer. The exhaust gas sample was dried prior to analysis. The technical specifications of the emission analyzer are given in Table 2. An ASTM D445 (Anton Paar, Austria) and ASTM 93 were used to measure viscosity and flash point, respectively. The details of the engine test are tabulated in Table 3.

**Table 2:** Specifications of the exhaust gas analyzer

Gas	Measuring test	Resolution	Accuracy
CO	0–4000 ppm	1 ppm	±5% of reading or ±10 ppm
HC	0–10% vol	0.01% vol	±0.3% of reading
NO_*x*_	0–4000 ppm	1 ppm	±5% of reading or ±5 ppm
Stack temperature	−20–1315 °C	1 °C	±2 °C
Probe tip temperature	800 °C max	—	—

**Table 3:** The details of the engine test

Make & Model	Kirloskar TAF1
Type: Four stroke	Single cylinder,
Direct injection	Bore X stroke (mm): 87.5 × 110
Compression ratio	17.5 : 1
Engine capacity	0.661 litre
Rated power	4.4 kW
Rated power rated speed	1500 rpm (constant speed)

The experimental uncertainties in the presented results were evaluated according to the root sum square method. Table 4 gives the uncertainties of the various measuring devices used in the present study.

**Table 4:** Uncertainties of experimental measurements

Instrument	Range	Accuracy	Uncertainty
Torque indicator, N m	0–200	±1% of reading	1
Fuel burette, cc	153	±0.2	1
Speed sensor, rpm	0–10000	±5 rpm	0.1
CO, ppm	0–4000	±10 ppm	1
UHC, % vol	0–10% vol	±0.3% of reading	0.1
NO_*x*_, ppm	0–4000	±5 ppm	1
Pressure transducer, bar	250	±1% of reading	1
Crank angle encoder, degree	0–720	±0.5	0.3
Brake power	—	—	±1

### 2.5 Desulfurization activity and reusability of the catalyst

The isodiesel and heavy diesel (Markazi Province, Iran) were selected as the feedstocks. Table 5 shows the main characteristics of the feedstocks. The reactions were performed in a fixed-bed microreactor. In a glass reactor, the experiments were directed in batch with a 20 kHz ultrasonic processor. A specific amount of model fuel (100 ml) and hydrogen peroxide 30% were added to the reactor. To maintain temperature, a water bath was employed. After maintaining the selected temperature, the mixture was sonicated for 15 min. After cooling at room temperature, the mixture was centrifuged for 10 min. The main characteristics of the product and feedstock were evaluated *via* the analytical methods: analysis of sulfur compound was performed using an X-ray sulfur analyzer (Korea). For industrial applications, the regeneration unit of the catalyst is important in selecting the catalyst for the ODS process. In the current study, the regeneration ability of the catalyst was investigated at elevated temperature (600 °C) in the presence of N_2_ gas. In the end of the reaction, the liquid feed containing the catalyst (1 g L^−1^) is pumped up to the reactor, and the catalyst was recovered by filtration, washed with acetonitrile and calcined at 600 °C.

**Table 5:** Results of the viscosity and flash point tests for fuels with and without additives

Properties	Iso diesel	Heavy diesel	Concentration of the nanostructure
Viscosity (40 °C) (mm^2^ s^−1^)	5.3	11.2	0 ppm
	6.72	12.33	1% by volume
Flash point (°C)	126	167	0 ppm
	134	175	1% by volume

## 3. Results and discussion

### 3.1 Structural analysis

Fig. 2 shows the XRD patterns of the as-made NiFe_2_O_4_ samples. As mentioned in Table 1, in a constant stoichiometric amount of cationic sources, the ratio of fuel was changed. In the sample without lactose, sample 1, the XRD pattern is not highly crystalline and only three peaks grow at 2*θ* equal to 35.97°, 44.12°, and 63.06° (Fig. 2a). Upon an increase in lactose concentration, the diffractograms contain peaks as compared to sample 1. These patterns match JCPDS 44-1485. However, Fe_2_O_3_ and NiO appear along with the major phase (Fig. 2b and c). The amounts of impurities are more obvious in sample 2 (Fig. 2b) as compared to sample 3 (Fig. 2c). The major phase in sample 2 grows at 30.45°, 35.85°, and 63.10°. The diffraction peaks of sample 3 and sample 4 are more intense as compared to sample 1. Fig. 2d and e show pure spinel NiFe_2_O_4_ and the diffraction peaks are observed at 30.36°, 35.70°, 43.40°, 57.36°, and 63.01°. The average crystallite size was calculated by Scherrer equation eqn (1),

1 *D* = *kλ*/*β* cos *θ*,

which was obtained to be around 3.8, 5.0, 16.7, 27.6, and 15.2 nm, for the samples 1, 2, 3, 4 and 5, respectively. In the Scherrer equation, *D* is the size of nanoparticles, *λ* is the X-ray wavelength, *β* is the line broadening at half the maximum intensity, *k* is a constant and *θ* is the diffraction of the peak.

**Fig. 2:**
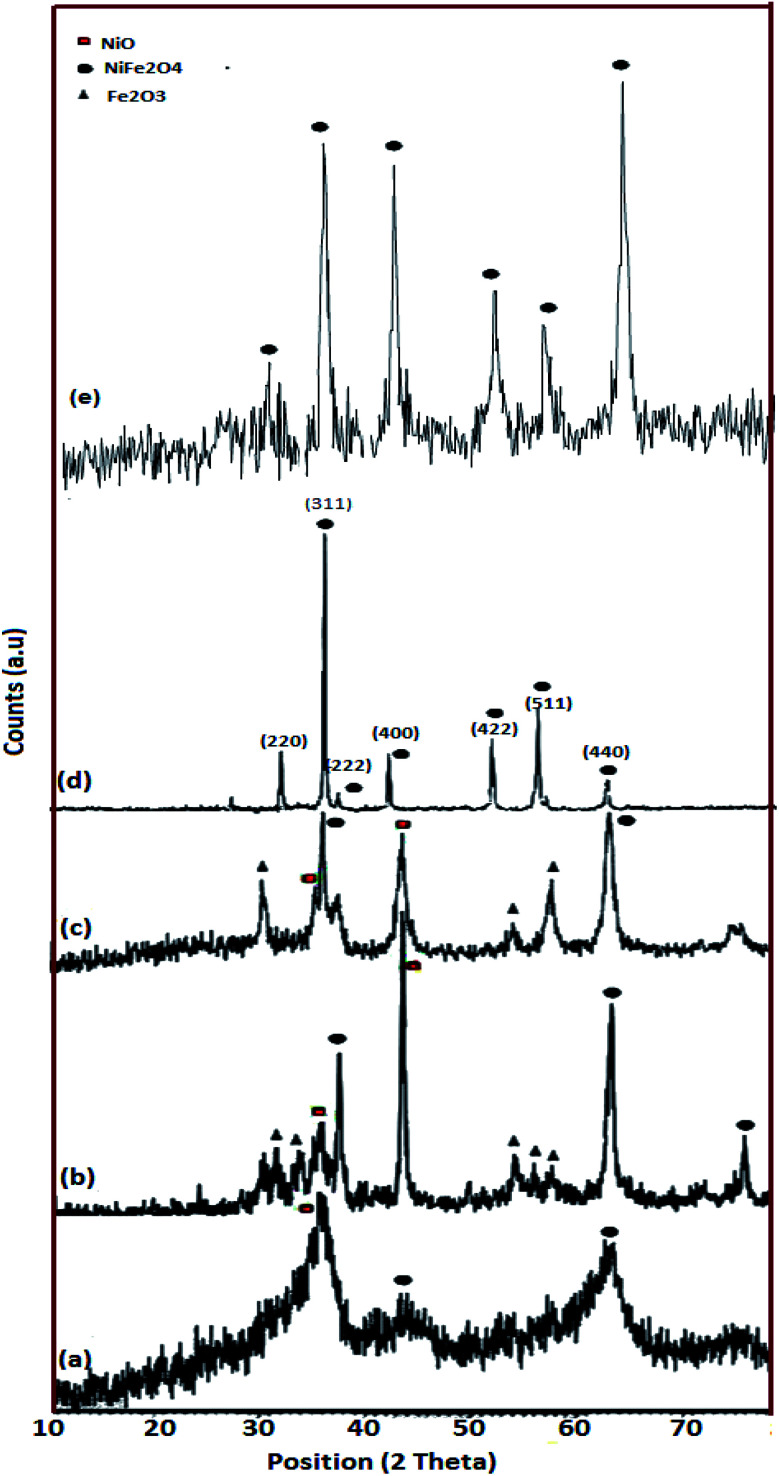
XRD patterns of the as-prepared NiFe_2_O_4_; (a) sample 1 (NiFe_2_O_4_ prepared without lactose), (b) sample 2 (NiFe_2_O_4_ prepared with 1.5 mol ratio lactose), (c) sample 3 (NiFe_2_O_4_ prepared with 3 mol ratio lactose), (d) sample 4 (hormone treated NiFe_2_O_4_) and (e) sample 5 (NiFe_2_O_4_ prepared with 6 mol ratio lactose).

### 3.2 Morphology and physico–chemical properties of hormone treated NiFe_2_O_4_ nanosheets

**Fig. 3:**
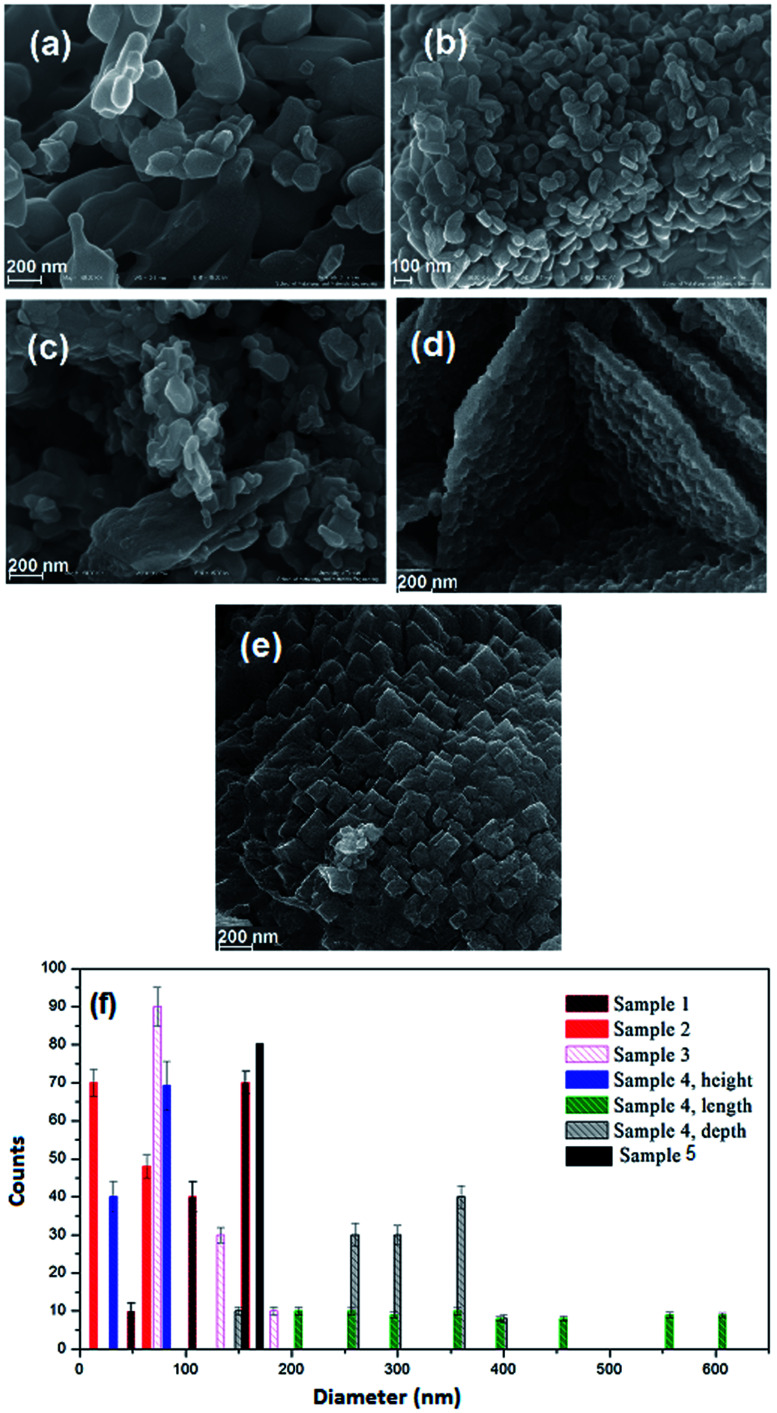
FESEM micrographs of NiFe_2_O_4_ prepared in various reductant : nitrate ratios; (a) sample 1 (NiFe_2_O_4_ prepared without lactose), (b) sample 2 (NiFe_2_O_4_ prepared with 1.5 mol ratio lactose), (c) sample 3 (NiFe_2_O_4_ prepared with 3 mol ratio lactose), (d) sample 4 (hormone treated NiFe_2_O_4_), (e) sample 5 (NiFe_2_O_4_ prepared with 6 mol ratio lactose) and (f) their respective histograms.

The impact of reductant concentrations on the surface morphology and the particle size of the samples were investigated using FESEM (Fig. 3a–e). It is clear that in the sample without lactose, the majority of the particles are agglomerated (Fig. 3a). Upon increasing lactose ratio to 1.5 (Fig. 3b), semi-spherical particles were formed, in a homogeneous texture. Fig. 3c and d show the NiFe_2_O_4_ samples synthesized with lactose/nitrate molar ratios of 3 : 1 and 6 : 1, respectively. In the latter morphology, the particles agglomerate, while in the former, cuboid (plate) structures are obtained. Each molecule of T3 hormone contains functional groups. These functional groups are iodine, amine and carboxylic acid in which T3 contains three iodide groups. These functional groups may react with metal nitrates and create weak links to form complex ions. Under the ultrasonic conditions, high temperature and pressure induce the complexes to decompose into the NiFe_2_O_4_ nanocrystals. To reduce the Gibbs free energy,^35^ primary particles deposit on the larger crystal's surface to form homogeneous regular shapes. T3 hormone has an important role in the formation of the one-dimension growth. The long chain of T3 molecules is absorbed on the various crystal planes, and acts as some face inhibited function surfactant to help competitive growth to form a sheet like morphology. Morphological observation of hormone treated NiFe_2_O_4_ confirms the formation of very thin nanosheets (Fig. 3d). In Fig. 3e (sample without T3 hormone), a large quantity of cubic structure can be observed. The results from morphological observations are tabulated in Table 2. The grain size estimated from FESEM data was discerned to be larger than the one obtained from XRD analysis. This indicated that every grain was formed by the aggregation of several tiny ferrite nanocrystallites or nano-particles.

**Fig. 4:**
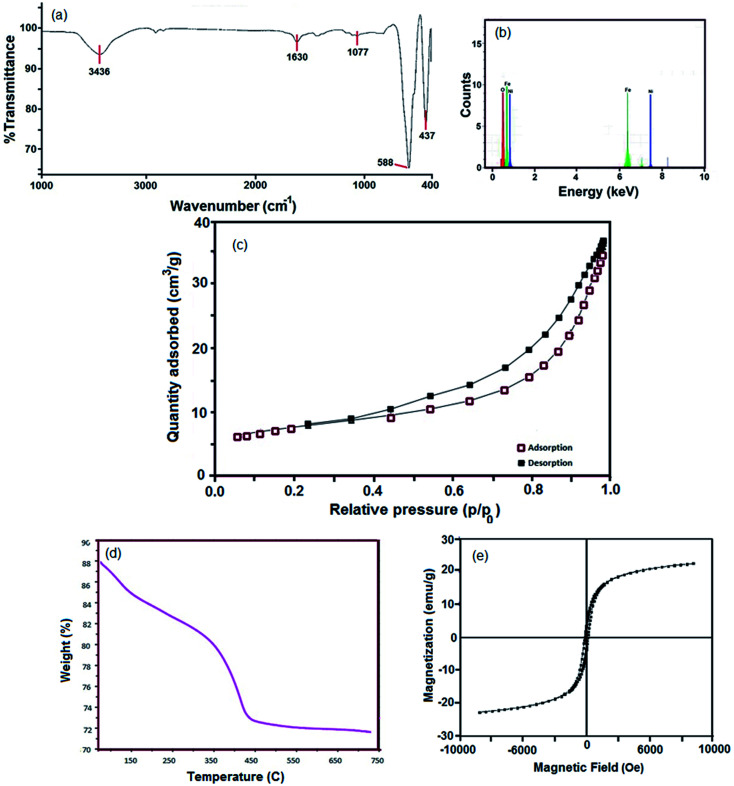
(a) FTIR spectrum of hormone treated NiFe_2_O_4_ nanosheets (sample 4), (b) EDS spectrum of hormone treated NiFe_2_O_4_ nanosheets (sample 4), (c) N2 adsorption/desorption isotherm, (d) TG analysis and (e) magnetization hysteresis loops of hormone treated NiFe_2_O_4_ nanosheets (sample 4).

The FTIR spectrum of the sample 4 (Fig. 4a) shows two intense bands at 588 cm^−1^ and 437 cm^−1^, which are related to the stretching vibration of Fe–O and Ni–O, respectively. The bands at 3436 cm^−1^ and 1630 cm^−1^ are due to the moisture (H–OH).^36^ The EDS analysis clearly affirms the presence of Ni, Fe and O in the hormone treated NiFe_2_O_4_ composition (Fig. 4b).

The NiFe_2_O_4_ hormone treated sample was selected in order to investigate the physico–chemical properties of this sample. Primarily, specific surface area was determined using Brunauer–Emmett–Teller (BET). The N_2_ adsorption/desorption isotherm can be observed in Fig. 4c. The obtained average pore diameter and specific pore volume of the NiFe_2_O_4_ nanosheets were 9.53 nm and 7.62 cm^3^ g^−1^, respectively. A porous structure with a BET surface area of 24.2 m^2^ g^−1^ was obtained, which can be classified as isotherm type IV and H3-type hysteresis. The H3-type hysteresis loop confirms mesoporous materials according to the IUPAC classification. The H3-type hysteresis shows the random distribution of pores. As a result, there is a significant increase in the BET surface area compared to other studies.^37^

The T3 treated sample powder was subjected to simultaneous thermogravimetric analysis (TGA) (Fig. 4d). The sample decomposed in three steps, 50–150 °C, 170–300 °C and 340–750 °C. The first weight loss region from 50 °C to 150 °C is related to the dehydration of water in the precursor sample. The weight loss at 170–300 °C could be due to the oxidation of organic materials and the third weight loss region represents the crystallization or phase formation process of NiFe_2_O_4_.

VSM is a standard method to evaluate the magnetic properties of the hormone treated sample. Fig. 4e depicts the magnetic hysteresis loop. The saturation magnetization (*M*_s_), remanence magnetization (*M*_r_) and coercivity (*H*_c_) values for the sample are 1.22 emu g^−1^, 0.08 emu g^−1^ and 80 Os respectively. Therefore, the NiFe_2_O_4_ nanostructure shows ferromagnetic behavior. The magnetism of the catalyst plays an important role in separation of the catalyst after the desulfurization process.

**Fig. 5:**
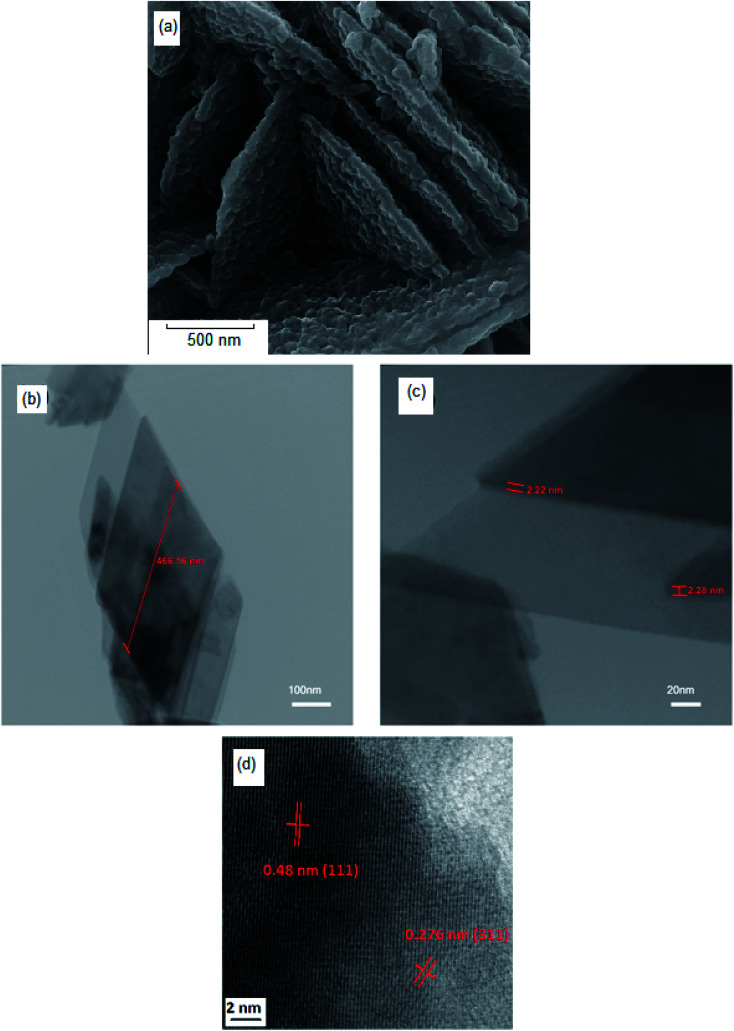
(a) SEM image of hormone treated NiFe_2_O_4_ (sample 4), (b and c) TEM images, and (d) high-resolution TEM (HRTEM) of hormone treated NiFe_2_O_4_ (sample 4).

3D observation of the sample (Fig. 5a–d) clearly shows that the NiFe_2_O_4_ formed with a nanosheet morphology with nanometer dimensions of around 466 nm × 2 nm. The corresponding high-resolution TEM (HRTEM) is shown in Fig. 5d, which shows the single-crystalline nature of these 2D nanosheets, with lattice spacings of 0.48 nm and 0.276 nm, in agreement with the (111) and (311) planes of NiFe_2_O_4_.

### 3.3 Catalytic activity

**Fig. 6:**
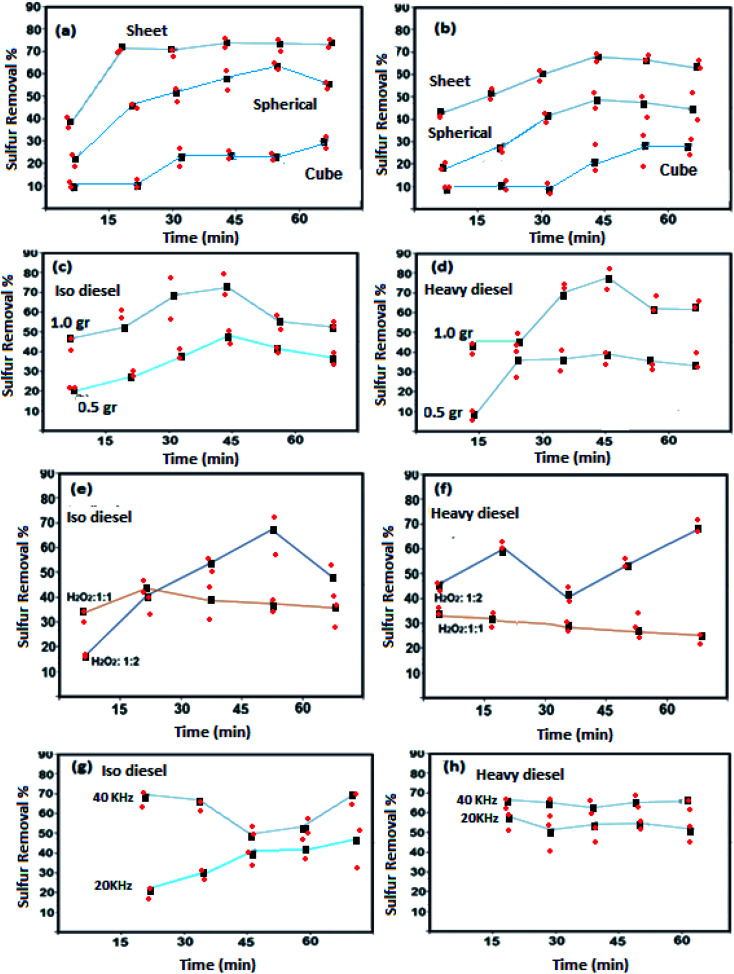
Sulfur removal *via* variation in catalyst (sample 3, sample 4 and sample 5) morphologies: (a) iso diesel and (b) heavy diesel, and sulfur removal *via* variation in absorbent (hormone treated NiFe_2_O_4_) doses: (c) iso diesel and (d) heavy diesel. Sulfur removal *via* variation in H_2_O_2_ doses in the presence of hormone treated NiFe_2_O_4_ as the catalyst: (e) iso diesel and (f) heavy diesel. Sulfur removal *via* variation in frequency in the presence of hormone treated NiFe_2_O_4_ as a catalyst: (g) iso diesel and (h) heavy diesel.

Oxidative activities of the synthesized NiFe_2_O_4_ nanostructure in different shapes of NiFe_2_O_4_ (semi-spherical and sheet) were studied with iso and heavy diesel as feedstocks and are shown in Fig. 6.

The significant changes in sulfur removal were observed during oxidative desulfurization by using different catalyst morphologies (Fig. 6a and b). Decomposition of sulfur compound can be influenced by basic or acidic sites present in the structure. The catalytic properties of NiFe_2_O_4_ nanostructures strongly depend on the nickel and iron ions and their distribution between the octahedral and tetrahedral sites that may enhance the extent of interactions of the phases at the border line during the desulfurization process. For instance, Ni and Fe are basic elements that can improve the decomposition of sulfur compound. The result indicates that the nanosheet morphology of NiFe_2_O_4_ has higher activity as compared to the other structures of NiFe_2_O_4_. This is most probably due to the high surface area of nanosheet morphology. Catalytic performances of cube NiFe_2_O_4_ (sample 5) are shown in Fig. 6. The results indicate that poor catalytic performance of cube-NiFe_2_O_4_ is due to a lower number of active sites on the surface of the catalyst, resulting in decreasing catalytic activity.^38^

Fig. 6c and d depicts that the sulfur removal from fuel can be increased on the basis of catalyst concentration with the nanosheet morphology from 0.5 to 1.0 g L^−1^. For isodiesel, the removal rates for 0.5 and 1.0 g L^−1^ were estimated to be around 45% and 70% at constant time (45 min), respectively. The number of active sites on the catalyst surface is directly related to the catalyst dose. The results reveal that the NiFe_2_O_4_ can be utilized as a new catalyst for sulfur removal.

Fig. 6e and f shows that the sulfur removal can be influenced by the concentration of H_2_O_2_. Increasing the concentration of H_2_O_2_ can be effective in removing sulfur compounds. For the two types of diesel fuels, the best results were obtained when the volume ratio of H_2_O_2_ to fuel is 1 : 2. Increasing concentration of H_2_O_2_ provides more oxidizing agents such as OH. Hence, desulfurization rate is greatly increased. During the ultrasonic process, H and OH radicals were generated by water pyrolysis. Different reactions to produce radicals are mentioned below:
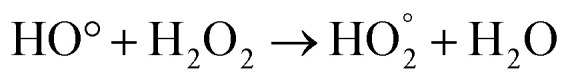
H_2_O_2_ + OH– HO_2_^−^ + H_2_O

Effects of ultrasonic frequency are described in Fig. 6g and h. It showed that useful results are obtained with a frequency of 40 kHz for the two types of diesel fuels. The elimination rate of sulfur compound reached 20.8% and 70.3% with frequencies of 20 and 40 kHz at 70 °C for 20 min of sonication in the system of H_2_O_2_–NiFe_2_O_4_-isodiesel, respectively. Sulfur removal rates were enhanced by an ultrasound probe in the UAOD process due to its smoother dispersion capability and high local temperatures and pressures.^39^

**Fig. 7:**
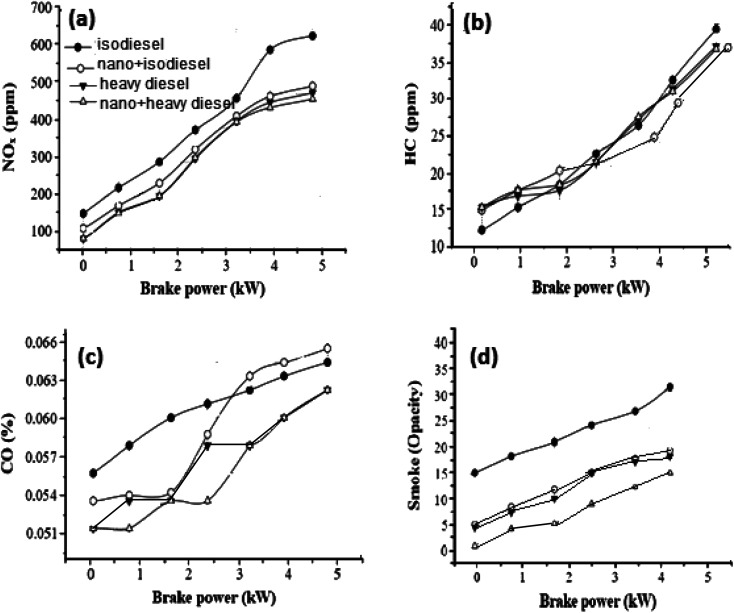
Gas emission in the presence of hormone treated NiFe_2_O_4_ (1%) for iso diesel and heavy diesel: (a) NO_*x*_, (b) HC, (c) CO and (d) smoke.

To improve the quality of diesel fuel, NiFe_2_O_4_ nanosheets were selected and doped in isodiesel and heavy diesel to reduce the amount of exhaust gases. Table 6 reports the results of various research papers on different nanofluid additives used in diesel to improve NO_*x*_ emission. By comparison, NiFe_2_O_4_ nanosheets + isodiesel and heavy diesel fuels induced a higher reduced level of NO_*x*_ emission during the combustion process (Fig. 7a). The NO_*x*_ emission in full load was 470 ppm, while it was 610 ppm for pure isodiesel. This shows that ferrite nanosheets act as a heat sink and reduce the cylinder temperature.^45^

**Table 6:** Engine performance by adding a nano additive in the diesel engine

Nano additive	Composition	NO_*x*_ emission %	Reference
Al	4%	>28	40
CNT	100 ppm	>30	41
Fe_3_O_4_	0.4%	>10	42
CeO_2_	2%	>7	43
Platinum/cerium	2%	>20	44

Fig. 7b shows the curve of HC emission with brake power. Hydrocarbon emission is the result of incomplete combustion of fuel. Most researchers reported that hydrocarbon emission is reduced by the addition of nano fuel additives because of higher evaporation rate and catalytic oxidation. Mehta RN^46^ studied the effect of nano aluminum as an additive. A decrease of hydrocarbon emission up to 4% was observed for this additive doped water-diesel fuel. The activity of NiFe_2_O_4_ nanosheets is comparable with that of others reported on the combustion process. Complete combustion and sufficient oxygen availability can be further improved with NiFe_2_O_4_ nanosheets compared to no additive. In the case of isodiesel, NiFe_2_O_4_ in 1% concentration showed a 27 ppm HC emission level, compared with the 32 ppm HC emission level of no additives.^47^

CO emissions for different brake powers are illustrated in Fig. 7c. Lower CO emission was obtained with the Ni based additive because the diesel fuels with metal-based additives were burned well in the cylinders than diesel fuel without metal-based additives. The CO concentrations at different brake powers with 1% nanosheets were decreased by 5% in comparison to isodiesel fuel without additives. NiFe_2_O_4_ nanosheets behave as an oxidation catalyst, leading to reduced ignition delay.^48^

From Fig. 7d, it can be concluded that the NiFe_2_O_4_ nanosheet with a dose level of 1% has been emphasized for its affirmative effect when mixed with isodiesel, resulting in a 10% reduction of smoke emission. The main reason for this result is the presence of the NiFe_2_O_4_ nano additive in the isodiesel and heavy diesel, which improves ignition characteristics.

Table 5 summarizes the properties of the feedstocks. The flash point is the lowest temperature that will produce sufficient vapor to provide a flammable mixture in the air. According to Table 5, an increase of 8 °C was observed in flash point after adding nanosheets at concentrations of 1% to isodiesel. By adding nano NiFe_2_O_4_, attraction forces between the particles increase and the flow from the liquid to gas phase reduces. The kinematic viscosity is important to study the clean diesel production process. On average there is about a 10% change in viscosity for the two kinds of diesel (Table 5), which can be attributed to the catalytic nanoparticle addition in fuel. The resistance between layers of fluid increases by adding the nanocatalyst, which in turn affects the viscosity. High viscosities can generate deposits and increase emissions of greenhouse gases.^49^

### 3.4 Regeneration of the catalyst

**Fig. 8:**
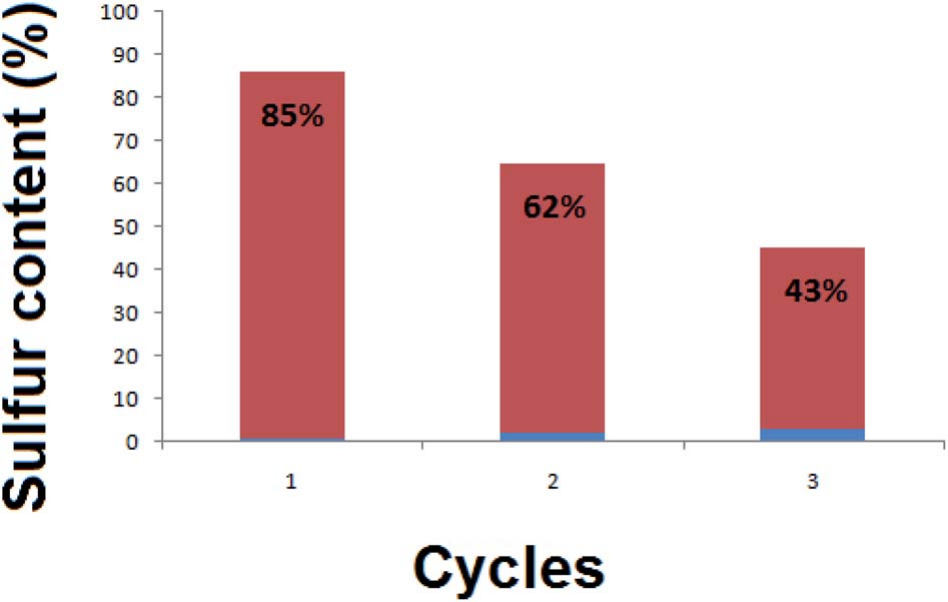
Recycling of the hormone treated NiFe_2_O_4_ (sample 4) after generation.

Fig. 8 presents three cycles of regeneration of the catalyst (NiFe_2_O_4_ nanosheets). From the first cycle to the third one, the sulfur capacity decreased. Normally, NiFe_2_O_4_ does not maintain its adsorption capacity which becomes nearly zero after 4 cycles.

**Fig. 9:**
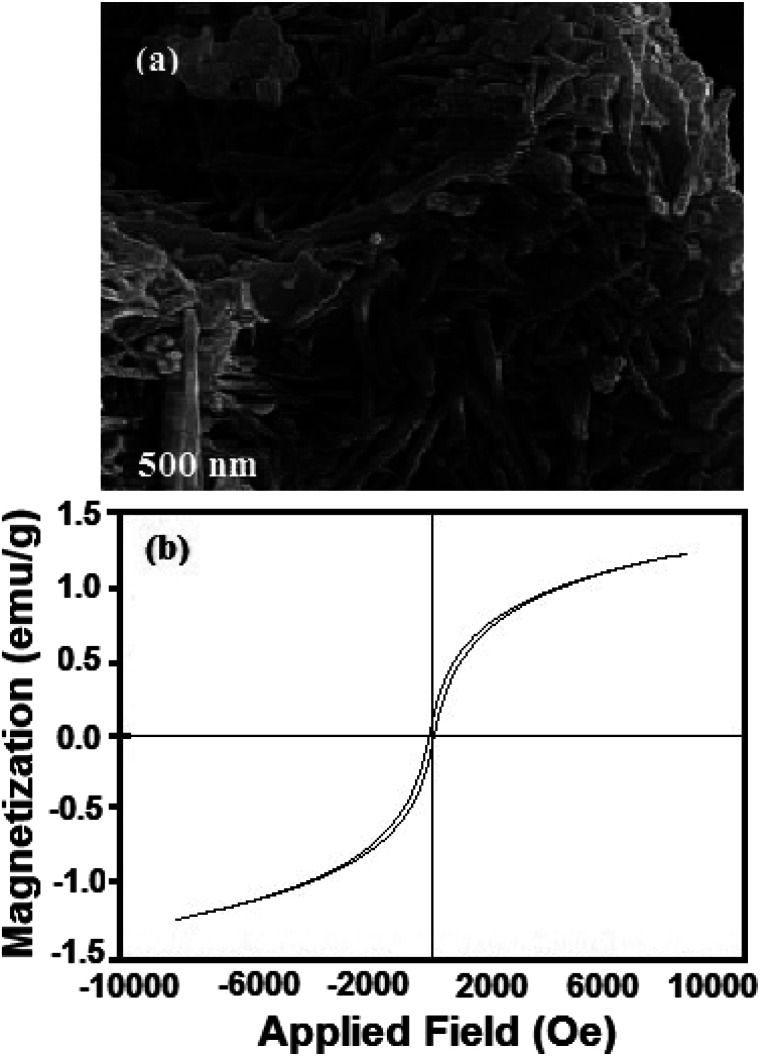
(a) FESEM micrographs of regenerated NiFe_2_O_4_ nanosheets and (b) magnetization hysteresis loop of the regenerated sample.

The regenerated NiFe_2_O_4_ was subjected to morphological and magnetic studies (Fig. 9). The FESEM micrograph (Fig. 9a) shows that the nanosheet morphology of the sample remains intact; however, it is less entangled as compared to the as-prepared sample (Fig. 3). The saturation magnetization (*M*_s_), remanence magnetization (*M*_r_) and coercivity (*H*_c_) values for the sample are 1.0 emu g^−1^, 0.05 emu g^−1^ and 78 Os, respectively. As compared to magnetization hysteresis of the as-prepared NiFe_2_O_4_ nanosheets (Fig. 4), the regenerated sample shows a 30% decrease in *M*_r_, 22% in *M*_s_, and 9% in *H*_c_ (Fig. 9b). The lower magnetization is due to the presence of an organic layer on the surface of NiFe_2_O_4_ nanosheets. The catalyst is attracted by an external magnetic field because of its ferromagnetic nature, which is important to form homogeneous systems.

## 4. Conclusions and future directions

NiFe_2_O_4_ nanostructures were synthesized by combined ultrasonic-combustion methods. Because of the unique chemical properties of NiFe_2_O_4_ nanosheets, T3 hormone (biotemplate) was used to control the shape and size. The physico–chemical properties of the hormone treated NiFe_2_O_4_ sample show that the as-prepared NiFe_2_O_4_ nanosheets have potential to be an efficient catalyst for the ODS process. Treatment of a real diesel led to 70% desulfurization under moderate conditions. Moreover, NiFe_2_O_4_ nanosheets are able to regenerate after quenching and this is one of the most critical factors to choose a right catalyst. To improve the engine performance an attempt is made and encouraging results were reported in detail. According to the analysis of our research paper, NiFe_2_O_4_ can be used as an additive in diesel due to its better combustion. Hence, the emission of noxious gases such as CO, HC, smoke and NO_*x*_ evidently decreases in isodiesel and heavy diesel. These advantages of NiFe_2_O_4_ nanosheets evidence their potential for environmental science and pollution research in the future. However, critical studies are in progress to trap the nanoparticles from the exhaust of the diesel engine.

## Nomenclature

CO: Carbon monoxide (ppm)
HC: Hydrocarbons (ppm)
HRTEM: High resolution transmission electron microscopy
FESEM: Field emission scanning electron microscopy
VSM: Vibrating sample magnetometer
NO_*x*_: Nitrogen oxides (ppm)
T3: 3,5,3′-Triiodo-l-thyronine
TMB: Tetramethylbenzidine
bTDC: Before top dead centre

## Conflicts of interest

The authors declare no competing interests.

## Supplementary Material
